# Impact of Occupational Exposure on Lead Levels in Women

**DOI:** 10.1289/ehp.7386

**Published:** 2005-02-09

**Authors:** Marija Popovic, Fiona E. McNeill, David R. Chettle, Colin E. Webber, C. Virginia Lee, Wendy E. Kaye

**Affiliations:** ^1^McMaster University, Hamilton, Ontario, Canada; ^2^Hamilton Health Sciences, Hamilton, Ontario, Canada; ^3^Agency for Toxic Substances and Disease Registry, Atlanta, Georgia, USA

**Keywords:** blood lead concentration, bone lead concentration, lead, occupational exposure, women’s health, X-ray fluorescence

## Abstract

In 1994, 207 women participated in a study designed to examine the effects of occupational exposure and various lifestyle factors on bone and blood lead levels. *In vivo* measurements of Pb concentrations in tibia were performed by X-ray fluorescence. All 108 former smelter employees and 99 referents provided blood samples and answered a questionnaire on lifestyle characteristics and the relevant medical history. Lead concentrations in tibia and blood were significantly higher in the exposed group. The difference in mean bone Pb concentrations of the two groups is markedly greater than the difference in the mean blood Pb concentrations, supporting the view that bone Pb measurements are a more reliable determinant of Pb body burden. Chronic exposure did not result in any statistically significant differences in adverse pregnancy outcomes. A significantly lower age at the onset of menopause in occupationally exposed women may suggest that Pb causes adverse changes in the pattern of estrus and menses. The exposed women had lower bone Pb concentrations than those found in most studies on predominantly male workers. Blood Pb concentrations remain increased in women long after the cessation of occupational exposure, reflecting the importance of the endogenous exposure. The endogenous exposure relation found for postmenopausal exposed women is consistent with data on male smelter workers, whereas the relation found for premenopausal women is significantly lower. This suggests that sex plays an important role in the metabolism of lead, and current models of exposure extrapolated from male data may be inappropriate for use on women.

The mobilization of endogenously stored lead represents an important health threat to individuals who have experienced elevated exposure in the past. From the skeleton as a primary storage site, lead gradually reenters the circulation through bone resorption. Endogenous exposure may be increased during phases of physiologic stress that are accompanied by elevated bone resorption. Various manifestations of Pb toxicity have been uncovered through epidemiologic investigations [World Health Organization (WHO) 1995]. Among the most harmful are its effects on the developing central nervous system of fetuses and children.

Calcium demands of the fetus and breast-fed infants are partially met through an increase in maternal bone turnover. Skeletal Pb is concurrently mobilized due to the similarities of the atomic and ionic structures of Pb and calcium. Maternal bone Pb levels are directly correlated with infant blood levels and umbilical cord levels of Pb (Hernandez-Avila et al. 2002; Sanin et al. 2001). In addition, the disturbance of skeletal homeostasis associated with menopause can result in a greater transfer of Pb from bone to the bloodstream. Although healthy men experience relatively constant life-long bone Pb accumulation with chronic exposure, exaggerated endogenous release is expected to occur in women with each pregnancy, throughout lactation, and during menopause.

The aim of this analysis was to uncover the differences between sexes in Pb metabolism. Cohorts of environmentally and occupationally exposed women were compared with regard to various medical and lifestyle factors, providing a degree of adjustment for confounders and more accurate identification of effects related to Pb exposure. Female former workers at the Bunker Hill smelter in Idaho were then compared with other, predominantly male, cohorts to investigate potential sex hormone-driven differences in the long-term skeletal accumulation of lead.

## Subjects and Methods

### Subjects and data collection.

In 1994, 108 women, former workers at the Bunker Hill smelter in northern Idaho, took part in a study conducted by the Agency for Toxic Substances and Disease Registry (ATSDR 1997). The women were enrolled on the basis of having worked at the plant for at least 30 days in the 1970s and had held a range of occupational duties. The exposure of the occupationally exposed women must have ceased by the closing date of the smelter in 1982, that is at least 12 years before the study. A cohort of referent women consisted of 99 women in the same age range as the exposed women and with no known occupational exposure to lead; they were recruited for the study based on the records from the Department of Motor Vehicles in the Spokane, Washington, area. Blood samples were analyzed by atomic absorption spectroscopy. Bone Pb measurements were taken with one of two ^109^Cd K X-ray fluorescence (XRF) instruments, one from the University of Maryland (Baltimore, MD, USA) and one from McMaster University (McNeill et al. 1999). The measurements were performed at the midpoint of the left tibia and the effective radiation dose from each measurement was 40 nSv.

Information on lifestyle characteristics and the relevant medical histories was determined from each woman’s responses to the questionnaire. The body mass index (BMI) was calculated as (mass ÷ height^2^) from the weight and height provided. Pregnancy history included information on the number of pregnancies, miscarriages, stillbirths, premature and normal births, and children born with defects. Although not explicitly defined in the questionnaire, some congenital defects reported by mothers included funnel chest, spina bifida, and a number of different heart conditions.

Years since cessation of menses was calculated and used to identify women with recent menopause (≤4 years). Women were further classified according to hysterectomy history. Women whose menses ceased due to hysterectomy were classified as having surgical menopause. The questionnaire did not differentiate between hysterectomies with and without ovariectomies. A crude distinction, which may have caused some misclassification, was made between the two by assuming that only hysterectomies with ovariectomies were followed by estrogen treatment.

All women were classified as current or former smokers or nonsmokers. Current and former smokers were asked for the age at which they started smoking and the number of cigarettes smoked during the period of highest smoking. Current smokers provided the number of cigarettes smoked per day at the time of the interview and one year before the study. Former smokers were asked how long they had smoked regularly and how many cigarettes they smoked daily while smoking most often. Similar information was obtained for alcohol consumption, but the questionnaire did not include information about daily alcohol intake or the type of alcoholic beverages consumed. Data on the duration of estrogen treatment and calcium and vitamin supplementation were also available for analysis. The amount of physical activity was determined from the number of sessions of exercise per week, the length of each session, and the number of years of regular exercise. A full description of the study has been previously published (ATSDR 1997).

### Statistical analysis.

The concentrations of heavy metals in tissues often exhibit left-skewed distributions; therefore, nonparametric analysis of blood and bone Pb concentrations (blood Pb in units of micrograms per deciliter whole blood and bone Pb in units of micrograms per gram bone mineral) is required. Women were dichotomized based on the yes/no answers recorded in the interviews. We used the Mann-Whitney test to investigate the equality of two population medians. Blood Pb is presented in terms of geometric means and geometric SDs, whereas inverse variance weighted average is used for bone Pb.

We used Spearman’s rank order correlation analysis to investigate the dependence of blood Pb and bone Pb on the following factors: age, BMI, drinking and smoking habits, pregnancy history, current and former use of estrogen, calcium and oral contraceptives, duration of each treatment, and the degree of physical activity. We used partial correlation analysis to investigate the influence of age on the relationship between blood Pb or bone Pb and other temporal variables. A *p-*value of ≤ 0.05 was considered statistically significant.

Further statistical manipulation required an assumption that data were normally distributed. We used best subsets regression to screen variables and identify multivariate models that describe the variations in blood Pb and bone Pb. Based on the nature of the conducted study, only a subset of recorded data was used in the multiregression models. Certain questions, such as the former smoking habits or age at menopause, did not apply to the entire group and were thus excluded from general models. The criterion for inclusion of a potential correlate in a model was the availability of at least 200 of the possible 207 data points. The fit was optimized by the least-squares method, and different models were compared based on the value of *R*^2^ adjusted for the number of predictors (*R* ^2^-adj). We used the variance inflation factor (VIF) to indicate the presence of multicollinearity among the predictors with a cutoff value of 4. All statistical analyses were performed using Minitab statistical software (Minitab 2000).

## Results

### Characteristics of the sample.

Table 1 summarizes the main characteristics of the referent and exposed groups. According to the Mann-Whitney test, the referent and exposed groups do not differ in age, but the BMI of the exposed women is higher (32.7 vs. 31.1, *p* < 0.05). Both blood Pb and bone Pb are significantly higher for the exposed cohort (*p* < 0.001).

Negative values of bone Pb recorded in some subjects arose from the subtraction of the background signal from the spectrum in the XRF analysis, as described by McNeill et al. (2000). Such values were retained in all analyses. We found the uncertainties in the XRF values (ΔXRF) to be unrelated to the magnitude of the XRF value. ΔXRF is correlated with the BMI (*p* < 0.001) and age (*p* < 0.05). A higher BMI factor results in attenuation of the X-ray signal because of a greater thickness of tissue overlying the tibia.

The mean age at menopause (surgical and natural combined) was lower for the exposed group (*p* = 0.001). This is in part a direct consequence of a significantly higher number of hysterectomies in the exposed group (*p* = 0.012), as 44.4% of the exposed and 23.2% of the referent women underwent hysterectomy at the mean age (± SD) of 33.0 ± 1.3 and 35.8 ± 1.3 years, respectively. However, when only natural menopause was considered, the mean age at menopause was still lower in the exposed group (43.7 ± 1.3 vs. 51.8 ± 1.1 years; *p* = 0.070). Among exposed and referent post-menopausal women, respectively, 74.2% and 50.0% reported having experienced surgical menopause. No distinction was made in the questionnaire between hysterectomies with and without ovariectomies. It is likely that only hysterectomies with ovariectomies were followed by estrogen treatment, and this occurred in 58.7% and 70.0% of all surgeries in the exposed and referent groups, respectively. With the exception of one woman, all examinees reported a medical reason for undergoing hysterectomy. However, this evidence is anecdotal and may not be reliable.

The exposed group is further characterized by more women who reported having at least one premature birth, miscarriage, or stillbirth (Table 2). The difference between the exposed and referent groups, however, is not statistically significant for any of the reported birth outcomes. Similarly, no statistically significant differences were noted at the *p* < 0.05 level between the two groups when birth outcomes reported by the same mother were considered as independent (Table 1). Higher numbers of premature births (*p* = 0.080) and stillbirths (*p* = 0.063) were reported in the exposed group. Pregnancy outcomes were further analyzed in relation to current and former smoking habits. We found no statistically significant differences between the groups of nonsmokers and smokers in either the referent or exposed group.

Factors including occupational exposure that statistically show a strong influence on mean bone Pb and blood Pb are listed in Table 3. Higher mean bone Pb is associated with smoking (ever/never) only in the exposed group. Higher bone Pb was associated with the use of estrogen (present or former) in both the whole referent group and the post-menopausal women in the referent group. Referent women who had never used oral contraceptive pills also had higher mean bone Pb. Significantly higher blood Pb was noted in smokers in the exposed group and in post-menopausal women belonging to either the exposed or referent group. When the cohort of postmenopausal women was divided into two groups, surgical menopause and natural menopause, exposed women who had not had surgery showed higher blood Pb. Referent women who used estrogen had significantly higher blood Pb, as did those who had never taken oral contraceptive pills.

Where the responses to the questionnaire allowed, we analyzed bone Pb and blood Pb to assess the correlation with temporal or quantitative variables. The resulting Spearman’s regression coefficients and the *p*-values of statistically significant relationships are summarized in Table 4. The analysis of partial correlations showed that most relationships of blood Pb and bone Pb with other temporal variables cannot be considered to be independent of age.

**Figure 1:**
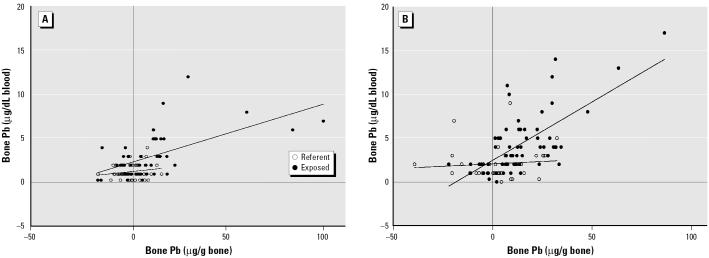
Endogenous release of Pb in premenopausal (*A*) and postmenopausal (*B*) women.

We expected the endogenous release of Pb from the skeleton into the blood stream to be reflected in an increase in blood Pb with bone Pb. We investigated the blood Pb versus bone Pb relationship separately for premenopausal and postmenopausal women because we expected menopause to be a major factor affecting bone Pb metabolism. No significant relationship was found in referent women, but we observed a strong positive correlation in the exposed women, as shown in Figure 1. The results suggest that the endogenous release rate (micrograms Pb per deciliter blood ÷ micrograms Pb per gram bone) in post-menopausal women is double the rate found in premenopausal women (0.132 ± 0.019 vs. 0.067 ± 0.014).

### Results of multivariate analyses.

We performed multivariate analysis to determine significant predictors of bone Pb and blood Pb. Separate multivariate regression models were formed for the exposed and referent groups. According to the values of VIF, the degree of collinearity among the independent variables was acceptable. Potential predictors, significant correlation coefficients, *p-*values, and the adjusted *R*^2^ are presented in Table 5. Blood Pb of the referent group is positively correlated with age, bone Pb, and alcohol consumption, whereas it seems to decrease with the number of pregnancies. Bone Pb of women in the referent group is positively correlated with blood Pb but not with age. A decrease in bone Pb appears to be associated with alcohol consumption and estrogen treatment. Multiple linear regression for exposed women confirmed that the blood Pb increases with bone Pb and also with the number of pregnancies. Age is not a direct predictor of blood Pb in exposed women. It is, however, strongly correlated with both bone Pb and the number of pregnancies, according to the results of bivariate analysis. The only statistically significant correlate of bone Pb in exposed women was blood Pb.

## Discussion

Women who had been occupationally exposed to Pb had significantly higher blood Pb and bone Pb than referent women. Bivariate and multivariate analysis showed that past occupational exposure was the predominant parameter explaining these differences, compared to many lifestyle factors.

The contribution of current environmental exposure to blood Pb can be estimated in two ways. Among premenopausal referent women, the correlation between blood Pb and bone Pb was insignificant, so the endogenous contribution to blood Pb was minimal. The average blood Pb in these subjects, 1.04 ± 0.09 μg/dL, provides one estimate of the exogenous contribution to blood Pb. Second, there was a significant correlation between blood Pb and bone Pb among exposed subjects, both premenopausal and postmenopausal women. For these two subgroups, the intercept in the endogenous relationship provides an estimate of blood Pb for zero bone Pb, that is the exogenous contribution to blood Pb. These values were 2.18 ± 0.35 μg/dL for premenopausal women and 2.52 ± 0.43 μg/dL for postmenopausal women. These levels are low compared to similar estimates derived from other studies (Table 6). The exception is the study by McNeill et al. (2000), which included groups of young men and women who were exposed as children when living in the vicinity of the Bunker Hill facility. These findings all confirm low current exogenous exposures of women who participated in this study.

The mean bone Pb among the occupationally exposed women was also low compared to values reported from previous studies (Table 7). In our study all of the subjects were female, whereas the large majority of exposed workers in other studies were male. However, we cannot conclude that the low levels found here were due to a sex difference either in Pb uptake or in Pb retention because the data are not available to compare either duration or intensity of exposure between studies.

A clear sex-related difference in the way the body handles Pb is demonstrated by the endogenous exposure relationship shown in Table 6. For different groups of men, the slope of the relationship between current blood Pb and tibia Pb in terms of micrograms Pb per deciliter blood ÷ micrograms Pb per gram bone mineral varied between 0.12 and 0.17. For the exposed young women described by McNeill et al. (2000), this slope was 0.052. For our premenopausal women the slope was 0.067; however, for our post-menopausal women the slope was 0.13. Therefore, young or premenopausal women retain Pb more avidly or release Pb more slowly than do men, whereas this distinction is lost for postmenopausal women. This is potentially associated with low estrogen levels in postmenopausal women. These data suggest that there is a need for sex-specific treatment of Pb exposure and metabolism studies.

The half-life of Pb in tibia is approximately 27 years in healthy men (Gerhardsson et al. 1993). Age-dependent increase in bone Pb is a marker of cumulative exposure that we expected to result in a similar increase in blood Pb through greater endogenous exposure; the bivariate analysis confirmed this expected pattern. Due to a strong influence of other factors, the multiregression model has shown age as positively correlated with blood Pb only in referent women. We found no significant difference in mean blood Pb and bone Pb in women with no prior pregnancies, possibly because of the low number of nulliparous postmenopausal women in this population. Blood Pb and bone Pb were positively correlated with the number of pregnancies in the exposed group. This is an artifact produced by a strong correlation between the number of pregnancies and age and, thus, the years of occupational exposure to lead. Although the bivariate analysis did not show a significant correlation between pregnancies and blood Pb of the referents, the multivariate regression resulted in a negative linear dependence between the two.

The adverse effects of Pb exposure on pregnancy outcome have been reported in several studies, including increased risk of spontaneous abortion (Borja-Aburto et al. 1999) and an increase in preterm births and stillbirths (McMichael et al. 1986). In our cohort, the total reported number of pregnancies was greater for the exposed group (3.18 vs. 2.76 pregnancies per woman), and 77.6% and 80.2% of pregnancies resulted in live births for the exposed and referent groups, respectively. Although the difference is not statistically significant at the *p* < 0.05 level, the exposed group is characterized by a greater reported number of stillbirths (*p* = 0.063) and infants born prematurely (*p* = 0.080) which may be suggestive of adverse effects of Pb on the mother and fetus (Tables 1 and 2).

Blood Pb in postmenopausal women was significantly higher (Table 3), which is consistent with results from other studies on Pb metabolism and general changes in bone metabolism associated with postmenopausal estrogen deficiency (Chapurlat et al. 2000; Ruegsegger et al. 1984; Silbergeld et al. 1988). Perimenopausal and postmenopausal bone loss can result in increased release of Pb into the blood stream, consistent with the notion that physiologic conditions which cause mobilization of bone calcium also cause mobilization of bone lead. Bone Pb of pre-menopausal and postmenopausal women did not differ significantly.

To our knowledge, premature menopause in humans due to prolonged exposure to Pb has not been investigated. This phenomenon was inferred in rhesus monkeys at subclinical exposure levels by Laughlin et al. (1987). The mean age (± SD) of menopause among the referent women in our study is 42.2 ± 1.3 years and that of the exposed women is 35.2 ± 1.3 years, compared with the average age of menopause of 51 years among U.S. women. We noted that 50.0% and 77.4% of women in the referent and exposed groups, respectively, have undergone surgical menopause. If only natural menopause is considered, the mean age at menopause is 51.8 ± 1.1 for referent women and 43.7 ± 1.3 for exposed women. Our analysis shows that among the exposed post-menopausal women, the mean blood Pb of women who underwent surgical menopause is lower than the mean of those who did not have the surgery. This presumably is a result of a smaller amount of Pb in the skeleton due to the longer period of higher bone turnover associated with the loss of endogenous estrogen.

**Figure 2:**
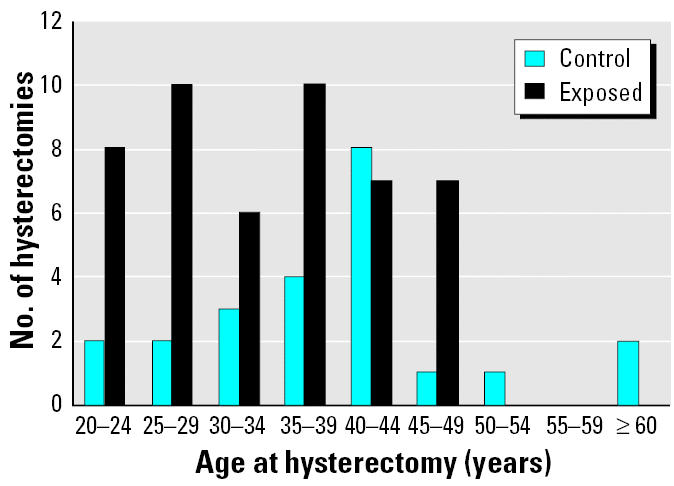
The numbers of exposed and referent women sorted by the age at which hysterectomy was performed.

According to the questionnaire data in this study, nearly one-half of the exposed women (mean age 46.3 ± 1.2 years) had undergone hysterectomy before the age of 50. The reasons for hysterectomies, as stated by the participants in the interview, are decidedly anecdotal and cannot be considered reliable. However, women of reproductive capacity may not have been allowed to work in production at the Bunker Hill smelter, which would have resulted in preferential hiring and retention of sterile women. In the referent group, 20.3% of women underwent hysterectomy, and the proportion of hysterectomies performed at an earlier age is lower. The number of hysterectomy cases reported in the present study is remarkably high (Figure 2); the annual rate of hysterectomy in the United States for 1988–1993 was 5.5 per 1,000 women according to the National Hospital Discharge Survey (Lepine et al. 1997).

Bone Pb was significantly greater in post-menopausal referent women treated with estrogen. Duration of estrogen treatment was also positively correlated with bone Pb of both referent and exposed women, but age cannot be excluded as a confounder in bivariate analysis. These results are in agreement with other studies that have reported a reduction in bone resorption associated with the use of estrogen (Webber et al. 1995). An auxiliary variable was entered in the multiregression model to identify women who had used estrogen (ever/never). The results found by multivariate analysis do not agree with those of the bivariate analysis, further exemplifying the underlying dependence on age.

Oral contraception is another source of estrogen. The influence of oral contraception on bone Pb metabolism or blood Pb levels, to our knowledge, has not been explicitly investigated. Our analysis showed that referent women who reported using oral contraceptive pills had lower mean blood Pb and bone Pb. The effect of oral contraceptives on Pb metabolism may be indirectly inferred through its relationship with bone mineral density, but the recent cross-sectional studies in this area have yielded discrepant results. According to the results of a controlled study by Benerson et al. (2001), mean bone mineral density remained unchanged or increased with the use of oral contraceptives, depending on the type of contraceptive used. A cross-sectional study by Prior et al. (2001) suggests that, while supplementation with estrogen and progestin may help maintain bone density in postmenopausal women, it is associated with bone loss in women 25–45 years of age. In our study we found no significant difference in mean blood Pb and bone Pb between the group of women 25–45 years of age and the group of women older than 45 years of age, based on the use of oral contraceptives (results not shown).

Cigarette smoking (ever/never) was a significant determinant of both mean blood Pb and bone Pb in the exposed group. In the bivariate analysis, the number of cigarettes smoked per day was positively correlated with bone Pb of the referent group. Our findings do not indicate markedly higher blood Pb among current smokers as reported in other studies (Symanski and Hertz-Picciotto 1995). The lack of correlation may be a consequence of a rather low number of current smokers in the referent group (12.1%) and the reported number of cigarettes smoked daily (17 on average). Smoking was not prohibited in the Bunker Hill smelter. The adverse effects of smoking on pregnancy outcome are generally accepted and extensively studied. In this analysis we did not detect any statistically significant differences in the number of reported miscarriages, stillbirths, premature births, or birth defects between the smoking and non-smoking mothers in either the referent or exposed groups.

Several potential limitations of our study may have affected the analysis. According to National Institutes of Health (NIH) guidelines (NIH 1998), 29.3% and 34.3% of referent and exposed women, respectively, may be considered Class II obese. As such, the cohort may not be fully representative of a general female population. We did not investigate the length and the extent of the occupational exposure. Consequently, all the exposed individuals were treated as a single group, regardless of the number of years spent at the smelter or the relative occupational exposure they experienced. The records of environmental Pb exposure in the proximity of the smelter were not available because monitoring of Pb in air was not enforced. Any observed differences in response to occupational and environmental Pb exposure may, therefore, be attributed to a degree of exposure or different metabolic responses to Pb in women. The participants in the referent group were selected from a more urban area. As a result, this population was more educated and had a higher socioeconomic status, which may have resulted in lower exogenous exposure. In 1975, a policy was instituted in the Bunker Hill smelter that prevented women of childbearing capacity from working in production. Consequently, it is possible that greater numbers of women who had undergone menopause or had a hysterectomy were included in the exposed group. According to the ATSDR final report (ATSDR 1997), this did not appear to be the case, as only two women had undergone menopause before they went to work at the facility.

Skeletal Pb is an important marker of cumulative exposure to Pb and was used in this study to show the dependence of endogenous exposure on menopausal status. Our data indicate that there are important differences in Pb metabolism between sexes, reflected in lower endogenous exposure of premenopausal women and lower bone Pb concentrations than found in other studies of occupationally exposed men. Future longitudinal studies are needed to monitor the changes in bone Pb and blood Pb in occupationally exposed women and to isolate the effects of menopause, pregnancies, and lactation on bone Pb metabolism. Significantly lower age at menopause and hysterectomy trends uncovered in this group of former smelter workers certainly provide motivation for further epidemiologic studies.

## Figures and Tables

**Table 1:** Descriptive statistics of the referent and Pb-exposed cohorts.

	Referent	Exposed	*p*-Value
Subjects (no.)	99	108	
Age (mean ± SD)	45.7 ± 1.3	46.3 ± 1.2	NS
BMI (mean ± SD)	31.1 ± 1.3	32.7 ± 1.2	0.044
Hysterectomy cases [no. (%)]	23 (23.2)	48 (44.4)	0.001
Age at hysterectomy (mean ± SD)	39.6 ± 11.4	33.9 ± 8.0	0.042
Postmenopausal women [no. (%)]			
Natural menopause	20	16	
Surgical menopause	20 (50.0)	46 (74.2)	0.012
Age at menopause (mean ± SD)			
Natural	51.8 ± 1.1	43.7 ± 1.3	0.070
Natural and surgical	42.2 ± 1.3	35.2 ± 1.3	0.001
Recency of menopause [no. (%)]			
≤4 years	13 (32.5)	15 (24.2)	NS
> 4 years	23 (57.5)	45 (72.6)	
Unknown	4 (10)	2 (3.2)	
Surgical menopause followed by estrogen treatment [no. (%)]	14 (70.0)	27 (58.7)	NS
Ever pregnant [no. (%)]	87 (87.9)	98 (90.7)	NS
Nulliparous postmenopausal women [no. (%)]	6 (15.0)	2 (3.2)	0.053
Total pregnancies (no.)	274	343	
Total live births [no. (%)]	219 (80.2)	266 (77.6)	NS
Total full-term births [no. (%)]	211 (96.3)	245 (92.1)	0.042
Premature births [no. (%)]	5 (2.3)	14 (5.3)	0.080
Miscarriages (no.)	39	55	NS
Stillbirths (no.)	1	6	0.063
Birth defects (no.)	12	7	NS
Former oral contraception use [no. (%)]	73 (73.7)	80 (74.1)	NS
Former calcium use [no. (%)]	37 (37.4)	39 (36.1)	NS
Former estrogen use [no. (%)]	29 (29.3)	37 (34.2)	NS
Smoking [no. (%)]			
Current	12 (12.1)	40 (37.0)	< 0.001
Former	33 (33.3)	25 (23.1)	NS
Never	53 (53.5)	43 (39.8)	0.046
Alcohol use [no. (%)]			
Current	76 (76.8)	68 (63.0)	0.028
Former	16 (16.1)	30 (27.8)	0.041
Never	7 (7.1)	10 (9.3)	NS
Bone Pb concentration, μg/g bone mineral (mean ± SE)	3.22 ± 0.50	14.4 ± 0.50	< 0.001
Blood Pb levels, μg/dL (mean ± SD)	1.25 ± 2.10	2.73 ± 2.39	< 0.001

NS, not significant (*p* ≥ 0.10).

**Table 2:** Number (%) of women who have experienced a given pregnancy outcome.

Outcome	Referent	Exposed
Live birth	84 (96.6)	93 (94.9)
Full-term birth	83 (98.8)	93 (100)
Premature birth	4 (4.8)	9 (9.7)
Miscarriage	29 (33.3)	30 (30.6)
Stillbirth	1 (1.2)	5 (5.1)
Birth defect	10 (11.5)	6 (6.1)

*p*-Values for pairwise comparison of proportions indicate that differences between groups are not significant. Percentage values indicate the ratio of women having a given outcome to the number of parous women in the group.

**Table 3:** Geometric mean blood Pb (μg/dL whole blood) and inverse variance-weighted mean bone Pb (μg/g bone mineral) concentrations for statistically significant factors.

Criterion	Response	No.	Mean ± SD	*p*-Value
Occupational exposure (mean ± SD)				
Yes	Bone Pb	108	14.4 ± 0.50*^a^*	< 0.001
No		99	3.22 ± 0.50*^a^*	
Yes	Blood Pb	107	2.73 ± 2.39	< 0.001
No		98	1.25 ± 2.10	
Smoked ever/never				
Yes	Bone Pb: exposed	65	18.1 ± 0.61*^a^*	0.002
No		43	6.87 ± 0.86*^a^*	
Yes	Blood Pb: exposed	64	3.67 ± 12.0	< 0.001
No		43	1.76 ± 2.49	
Ever used estrogen				
Yes	Bone Pb: referent	29	7.73 ± 0.94*^a^*	0.003
No		70	1.41 ± 0.60*^a^*	
Yes	Blood Pb: referent	28	1.56 ± 2.21	0.038
No		70	1.15 ± 2.03	
Still have menstrual periods				
Yes	Blood Pb: referent	59	1.04 ± 1.97	0.002
No		39	1.65 ± 2.14	
Yes	Blood Pb: exposed	46	2.09 ± 2.49	0.012
No		61	3.33 ± 2.19	
Surgical menopause				
Yes	Blood Pb: exposed	47	2.87 ± 2.12	0.008
No		14	5.46 ± 2.02	
Postmenopausal women who have used estrogen				
Yes	Bone Pb: referent	24	9.18 ± 1.08*^a^*	0.007
No		16	1.83 ± 1.32*^a^*	
Ever used oral contraceptives				
Yes	Bone Pb: referent	73	1.39 ± 0.61*^a^*	0.036
No		26	7.22 ± 0.90*^a^*	
Yes	Blood Pb: referent	72	1.13 ± 2.00	0.006
No		26	1.66 ± 2.66	

Additional factors that were tested and did not show a correlation with Pb levels were: former smoking (smoke now), alcohol consumption (ever/never), former drinking (drink now), ever been pregnant, exercise regularly, and ever used calcium or vitamin supplements.

a Mean ± SE.

**Table 4:** Bivariate analysis: significance levels of Spearman’s rank correlation.

	Referent		Exposed	
Variable	Blood	Bone	Blood	Bone
Age (years)	0.01	0.05	0.02	
BMI (kg/m^2^)			0.01	
Time since menopause (years)				0.02
Time since hysterectomy (years)			0.01*^a^*	0.01
Duration of estrogen treatment (years)		0.05		0.05
Total no. of pregnancies, lifetime			0.01	0.05
Time since stopped using oral contraceptives (years)				0.05
Current smokers (cigarettes/day)		0.05		
Age when started consuming alcohol regularly (years)	0.01	0.01		
Age when quit consuming alcohol regularly (years)			0.05	
Regular exercise (years)			0.01*^a^*	
Exercise (times/week)			0.01	0.01
Cumulative time of regular exercise, lifetime			0.01	0.01

a The effect of age on the given variable does not reach the 5% level of significance; factors with *p*-values < 0.05 were duration of calcium and oral contraceptive use, time since started using oral contraceptives, number of cigarettes smoked daily 1 year before the study by current smokers, maximum number of cigarettes smoked daily by current and former smokers, number of years smoking by former smokers, and age when started smoking.

**Table 5:** Results of multilinear regression.

Variable	Coefficient	SE	*p*-Value	VIF
Referent				
Dependent variable: blood Pb	*R*^2^-adj = 31.4%			
Independent variable				
Bone Pb	0.031	0.011	0.009	1.3
Age	0.047	0.018	0.010	3.9
Alcohol consumption	1.008	0.434	0.023	1.2
No. of pregnancies	–0.127	0.060	0.037	1.2
Referent				
Dependent variable: bone Pb	*R*^2^-adj = 20.5%			
Independent variable				
Blood Pb	2.643	0.994	0.009	1.6
Alcohol consumption	–11.758	3.919	0.004	1.2
Estrogen therapy	–6.486	3.013	0.034	2.0
Exposed				
Dependent variable: blood Pb	*R*^2^-adj = 45.8%			
Independent variable				
Bone Pb	0.087	0.013	< 0.001	1.2
No. of pregnancies	0.285	0.127	0.028	1.4
Exposed				
Dependent variable: bone Pb	*R*^2^-adj = 36.4%			
Independent variable				
Blood Pb	3.872	0.574	< 0.001	1.4

Potential predictors of blood Pb and bone Pb with *p* > 0.05 were BMI, number of cigarettes smoked per day, still have menstrual periods (yes/no), use of birth control pills (ever/never), use of calcium supplement (ever/never), use of vitamin supplements (ever/never), and regular exercise (yes/no).

**Table 6:** Comparison of endogenous exposure rates with those found in other studies.

Reference	Exposure site (*n*)	Endogenous exposure (μg/dL)/(μg/g)	Baseline exposure (μg/dL)	Premenopausal women (Bunker Hill smelter)*^a^*	Postmenopausal women (Bunker Hill smelter)
Present study	Premenopausal women	0.067 ± 0.014	2.18 ± 0.35		
	Postmenopausal women	0.132 ± 0.019	2.53 ± 0.43		
McNeill et al. 2000	Environmentally exposed young men, USA (126)	0.126 ± 0.022	3.33 ± 0.25	*p* < 0.025	NS
	Environmentally exposed young women, USA (128)	0.052 ± 0.011	1.60 ± 0.12	NS	*p* < 0.001
Fleming 1998	Lead smelter, Canada				
	Active (204)	0.136 ± 0.014	13.6 ± 0.80	*p* < 0.001	NS
	Retired (14)	0.162 ± 0.051	6.10 ± 3.60	*p* < 0.05	NS
Cake 1998	Recycling plant, Canada				
	Active (49)	0.161 ± 0.055	29.5 ± 2.50	*p* < 0.10	NS
Bleecker et al. 1995	Lead smelter, Canada				
	Before strike (84)	0.120 ± 0.028	27.1 ± 1.33	*p* < 0.10	NS
	After strike (84)	0.170 ± 0.025	12.8 ± 1.18	*p* < 0.001	NS
Gerhardsson et al. 1993*^b^*	Lead smelter, Sweden				
	Retired (30)	0.133	5.27		
Erkkila et al. 1992*^b^*	Lead acid battery factory, Finland				
	Retired (16)	0.138	7.71		

NS, not significant at *p* < 0.1.

a *p*-Values express the statistical difference between the endogenous exposure rates.

b The uncertainties were not provided.

**Table 7:** Overview of mean tibial Pb concentrations determined in recent epidemiologic studies.

Study population	No.	Mean tibia Pb (μg/g; ± SD)	Range of bone Pb
Present study			
Environmentally exposed women	99 F	3.22 ± 0.50	^−^39.1–32.44
Former smelter workers	108 F	14.4 ± 0.50	^−^21.97–100.14
Nonoccupational exposure			
Environmentally exposed young men, USA (McNeill et al. 2000)	126 M	4.54 ± 0.31	
Environmentally exposed young women, USA (McNeill et al. 2000)	128 F	5.61 ± 0.43	
Nonexposed active workers, Sweden (Gerhardsson et al. 1993)	31 M	3.4	^−^9.4–13.3
Nonexposed retirees, Sweden (Gerhardsson et al. 1993)	10 M	12.0	^−^6.7–23.7
Nonexposed workers, Finland (Erkkila et al. 1992)	16 M, 10 F	3.5 ± 10.8	
Battery plant office workers, Finland (Erkkila et al. 1992)	19 M, 19 F	7.7 ± 11.3	
Nonexposed workers, UK (Somervaille et al. 1988)	12 M, 8 F	16.7 ± 3.7	
Active workers in Pb industry			
Primary Pb smelter workers, Canada (Bleecker et al. 1995)	84 M	40.0	^−^12–90
Primary Pb smelter workers, Sweden (Gerhardsson et al. 1993)	70 M	13.0	^−^4.1–72.8
Lead acid battery factory workers, Finland (Erkkila et al. 1992)	74 M, 17 F	21.1 ± 17.0	
Primary Pb smelter workers, Belgium (Roels et al. 1995)	123 M	49.0 ± 1.78	15.3–167.1
Precious metal smelter workers, UK (Somervaille et al. 1988)	15 M	54.8 ± 10.6	
Lead acid battery plant workers (Somervaille et al. 1988)	83 M, 5 F	32.3 ± 3.0	
Lead crystal glass factory (Somervaille et al. 1988)	81 M, 6 F	31.0 ± 3.4	
Retired Pb industry workers			
Primary Pb smelter retirees, Sweden (Gerhardsson et al. 1993)	30 M	39.3	2.9–73.4
Lead acid battery factory retirees, Finland (Erkkila et al. 1992)	12 M, 4 F	32.4 ± 34.9	

Abbreviations: F, female; M, male.
